# Correlation Between Blood Glucose Variability Indices Using Continuous Glucose Monitoring in Gestational Diabetes Patients and Abnormal Glucose Levels 42 Days Postpartum

**DOI:** 10.1155/jdr/1021066

**Published:** 2025-07-14

**Authors:** Rui Wang, Yuping Zhang, Huiqian Zeng, Jinyan Xiao, Mingming Qi, Lei Bao, Ruifen Jiao, Jing Liu, Yichun Li, Shuli He, Yunlong Li, Rui Li, Fan Ping, Yanping Liu

**Affiliations:** ^1^Clinical Nutrition Department, Peking Union Medical College Hospital, Beijing, China; ^2^Obstetrical Department, Guangzhou Women and Children's Medical Center, Guangzhou, China; ^3^Obstetrical Department, Yueyang Maternal and Child Health Care Hospital, Yueyang, China; ^4^Obstetrical Department, Xiangya Hospital Zhuzhou Central South University, Zhuzhou, China; ^5^Obstetrical Department, Xi'an People's Hospital, Xi'an, China; ^6^Obstetrical Department, Shijiazhuang Obstetrics and Gynecology Hospital, Shijiazhuang, China; ^7^Obstetrical Department, Guiqian International General Hospital, Guiyang, China; ^8^Obstetrical Department, The Second Hospital University of South China, Hengyang, China; ^9^Endocrinology Department, Peking Union Medical College Hospital, Beijing, China

**Keywords:** continuous glucose monitoring, gestational diabetes mellitus, glucose variability, postpartum OGTT, pregnancy outcomes

## Abstract

**Objective:** This study was aimed at analyzing the impact of blood glucose variability (GV) in gestational diabetes mellitus (GDM) patients on glucose outcomes 42 days postpartum and pregnancy outcomes. Additionally, it explored differences between various GV indices and evaluated their predictive values.

**Methods:** This retrospective study included 75 pregnant women diagnosed with GDM. Continuous glucose monitoring (CGM) was initiated postdiagnosis, and outcomes were followed up. Oral glucose tolerance tests (OGTTs) were conducted 42 days postpartum to assess glucose response.

**Results:** A total of 75 patients were included, among whom 8 (10.67%) exhibited impaired fasting glucose (IFG) and 7 (9.33%) impaired glucose tolerance (IGT) in the 42-day postpartum OGTT. No cases of diabetes were diagnosed. The results of the postpartum OGTT were significantly correlated with various GV indexes. In multivariate analysis, LBGI (OR: 1.437; 95% CI: 1.015–2.035; *p* = 0.041), *M* value (OR: 1.215; 95% CI: 1.030–1.434; *p* = 0.021), and TBR% (OR: 1.138; 95% CI: 1.020–1.271; *p* = 0.021) independently influenced IFG. Receiver operating characteristic (ROC) analysis indicated areas under the curve (AUCs) of 0.877 (95% CI: 0.760~0.994), 0.853 (95% CI: 0.730~0.975), 0.869 (95% CI: 0.748~0.991), and 0.793 (95% CI: 0.622~0.963) of IFG prediction model performance of TBR%, LBGI, *M* value, and HbA1c% combined with age, BMI, and family history of diabetes, respectively.

**Conclusion:** Blood GV is an independent factor influencing IFG 42 days postpartum in GDM women, especially with hypoglycemia. It can increase the predictive efficiency of the postpartum abnormal blood glucose prediction model.

**Trial Registration:** Chinese Clinical Trial Registry number: ChiCTR2100054833

## 1. Introduction

Gestational diabetes mellitus (GDM) is a common metabolic disorder first diagnosed during pregnancy, characterized by impaired glucose tolerance (IGT). It poses significant risks to maternal and fetal health, potentially leading to macrosomia, preterm birth, and neonatal hypoglycemia among other complications [1–3]. Research has shown that well-controlled blood glucose levels in GDM patients correlate with better pregnancy outcomes. However, despite HbA1c being the gold standard for assessing glucose control, it does not provide a complete picture of glucose dynamics. With advancements in modern technology, continuous glucose monitoring (CGM) offers a research perspective beyond traditional metrics and holds promise for establishing new management standards [4].

Blood glucose variability (GV) serves as a crucial supplement to describe glucose homeostasis. It is increasingly recognized in clinical practice as a significant indicator of glucose control [5]. Nevertheless, defining GV remains challenging, despite numerous proposed indices. There is currently no consensus on the definitive method for measuring GV in clinical practice and research. The latest ADA guidelines recommend CGM use and propose a 70% time in range (TIR) as a corresponding GV management standard for Type 1 diabetes patients [6]. However, the use of CGM in Type 2 diabetes mellitus (T2DM) and GDM patients remains controversial, with a lack of relevant research data and management standards. Therefore, this study is aimed at elucidating the relationship between CGM indices in GDM women and pregnancy outcomes, as well as glucose outcomes. By deepening our understanding of the role of GV in the 42-day postpartum glucose response of GDM women, we aim to clarify the significance of different GV indices in GDM management and analyze their potential predictive value.

## 2. Materials and Methods

### 2.1. Study Design

This study is a retrospective analysis of intervention group data from a large prospective multicenter randomized controlled trial. The original study intervention included nutritional education, counseling, and blood glucose monitoring including CGMs. For details on study design and specific interventions, please refer to the original research article (http://journal11.magtechjournal.com/Jwk_jcyxylc/CN/Y2024/V44/I4/434).

The study included data from seven subcenters from Jan 2022 to March 2024: Guangzhou Women and Children's Medical Center, Guiqian International General Hospital, Shijiazhuang Obstetrics and Gynecology Hospital, Xi'an People's Hospital, Yueyang Maternal and Child Health Care Hospital, The Second Hospital University of South China, and Xiangya Hospital Zhuzhou Central South University. Patients were diagnosed with GDM during routine antenatal care and obstetric records at gestational weeks 24–28 in each participating center. Ethical approval for this study was obtained from the Ethics Review Committee of Peking Union Medical College (Approval No. JS-3254).

Inclusion criteria were (1) aged 24–40 years, (2) confirmed diagnosis of GDM by OGTT, (3) singleton pregnancy, willing to participate and highly cooperative, (4) voluntary participation with informed consent signed by patients and family members, and (5) complete endpoint data available. Exclusion criteria were (1) pre-existing history of glucose abnormalities before pregnancy; (2) severe pulmonary, hepatic, renal, or other diseases; (3) abnormal thyroid function; and (4) history of mental illness.

### 2.2. Diagnosis Criteria

Patients were diagnosed with GDM according to the diagnostic criteria recommended by the American Diabetes Association guidelines [7]. Specifically, fasting, 1-h postprandial, and 2-h postprandial blood glucose thresholds were set at 5.1, 10.0, and 8.5 mmol/L, respectively. A result meeting or exceeding any of these thresholds constituted a diagnosis of GDM.

Abnormal glucose levels at 42 days postpartum were diagnosed based on the criteria from the Chinese guidelines for diabetes [8]. Specifically, impaired fasting glucose (IFG) was defined as venous fasting blood glucose levels between 6.1 and 7.0 mmol/L, with an OGTT 2-h blood glucose level ≤ 7.8 mmol/L. IGT was diagnosed when venous fasting blood glucose was < 7.0 mmol/L and OGTT 2-h blood glucose levels were between 7.8 and 11.1 mmol/L. Diabetes was diagnosed if venous fasting blood glucose was ≥ 7.0 mmol/L or OGTT 2-h blood glucose levels were ≥ 11.1 mmol/L.

### 2.3. CGM

Following the initial diagnosis of GDM at gestational weeks 24–28, this study employed two different real-time CGM systems: MicroTech Medical AiDEX (Hangzhou, Zhejiang, China) and GLU@U (Beijing, China). The MicroTech Medical AiDEX was used for Guangzhou Women and Children's Medical Center, Guiqian International General Hospital, Shijiazhuang Obstetrics and Gynecology Hospital, and Xi'an People's Hospital; GLU@U was used for Yueyang Maternal and Child Health Care Hospital, The Second Hospital University of South China, and Xiangya Hospital Zhuzhou Central South University. Participants had CGM sensors inserted subcutaneously into the posterior upper arm by physicians and calibrated according to standard operating guidelines. The MicroTech system recorded glucose levels every 5 min (range 20–250 mg/dL), while the GLU@U system recorded every 3 min (range 17–400 mg/dL). After 2 weeks, participants returned to the study site for sensor removal, and recorded data were downloaded and stored in an Excel database for further analysis. Participant's monitoring duration ranged from 119 to 670 h. The CGM target range was set at 3.5–7.8 mmol/L, in accordance with the updated 2019 standards for CGM.

### 2.4. Blood GV Assessment

Blood GV was assessed using the widely used EasyGV 9.0.R2 (Oxford University) [9] automated calculation program. The metrics generated by EasyGV included *M* value, mean amplitude of glycemic excursions (MAGEs), lability index (LI), average daily risk range (ADRR), *J*-index, low blood glucose index & high blood glucose index (LBGI & HBGI), continuous overall net glycemic action (CONGA), mean of daily differences (MODD), glycemic risk assessment diabetes equation (GRADE), and mean average glucose (MAG). According to Kovatchev and Cobelli [10], these GV indices were initially categorized into amplitude characteristics or time characteristics describing GV.

#### 2.4.1. Amplitude Characteristics Measuring the Amplitude of GV

##### 2.4.1.1. Average Amplitude

CV standard deviation divided by mean and multiplied by 100%

SD average deviation of glucose readings from the mean

*J*-index [11]a simple formula based on mean and standard deviation

##### 2.4.1.2. Extreme Amplitude

LBGI/HBGI [12]frequency and severity of hypoglycemic/hyperglycemic episodes

ADRR [13]summation of peak risks of hypoglycemia and hyperglycemia

GRADE [14]conversion of glucose values into risk scores, calculating the median

##### 2.4.1.3. Absolute Amplitude

MAGE [15]average amplitude of glucose fluctuations from peak to nadir

MAG [16]absolute difference between consecutive glucose readings divided by time

##### 2.4.1.4. Stability

LI [17]calculation of instability using three consecutive glucose values, averaged subsequently

*M* value [18]detection of differences between glucose and normal glucose

#### 2.4.2. Time Characteristics Measuring the Timing of GV

##### 2.4.2.1. Within-Day GV

CONGA [11]difference between current glucose readings and those from several hours ago

TIR [19]percentage of time spent within the target glucose range

Time above range (TAR) [19]percentage of time spent above the target glucose range

Time below range (TBR) [19]percentage of time spent below the target glucose range

##### 2.4.2.2. Between-Day GV

MODD [20]average absolute difference in glucose values at a specific time and those 24 h apart the previous day

### 2.5. Clinical Data Collection

Baseline data including patient demographics and family history were collected through postenrollment questionnaires. Postpartum, relevant pregnancy outcome data were extracted from hospital electronic medical records. Follow-up at 42 days postpartum included OGTT testing conducted at the hospital to collect relevant data.

### 2.6. Statistical Methods

Data were analyzed using SPSS 25.0 statistical software. Normality tests were performed on all continuous variables, and normally distributed data were described using mean ± standard deviation. Pearson correlation tests were used where appropriate; otherwise, data were described using quartile ranges, and Spearman correlation tests were conducted. Logistic regression was employed to determine if metrics were independent factors adjusted for other influencing factors. Receiver operating characteristic (ROC) curves were used to assess the predictive capabilities of models. Heatmaps were generated using Origin 2021 software. Statistical significance was set at *p* < 0.05.

## 3. Results

From May 2021 to April 2024, a total of 75 patients voluntarily enrolled in the study and provided informed consent. The average age of the pregnant women was 31.91 ± 3.40 years. The average prepregnancy BMI was 21.83 ± 2.59. The mean birth weight was 3215.53 ± 365.47 g, with one case (1.33%) of macrosomia. One newborn (1.33%) experienced hypoglycemia. At 42 days postpartum, OGTT results indicated that 8 patients (10.67%) had IFG, 7 patients (9.33%) had IGT, and no mothers were diagnosed with diabetes (Table 1).

**Figure 1:**
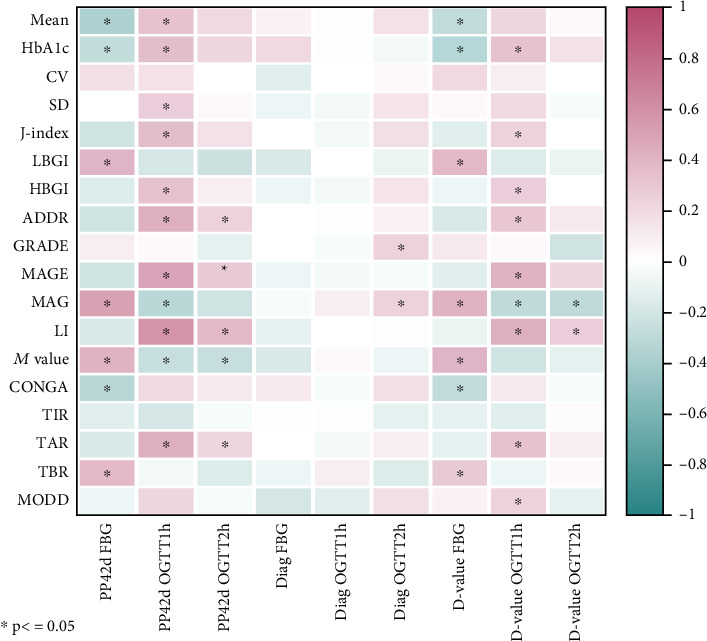
Heatmap of correlations between different GV metrics and OGTT results at diagnosis and 42 days postpartum. PP: postpartum period, Diag: diagnosis, *D* value: difference value, FBG: fasting blood glucose, OGTT: oral glucose tolerance.

To assess the correlation between GV metrics and the diagnosis of pregnancy outcomes and postpartum OGTT results, correlation analyses were conducted (Figure 1). There was no significant correlation observed between newborn weight and various GV metrics. Diagnosis of OGTT 2-h blood glucose levels showed positive correlations with GRADE and MAG, while other OGTT blood glucose values at diagnosis did not show significant correlations with GV metrics.

At 42 days postpartum, fasting blood glucose levels showed a significant positive correlation with LBGI, *M* value, MAG, and TBR and a significant negative correlation with mean, CONGA, and HbA1c. OGTT 1-h blood glucose levels at 42 days postpartum showed positive correlations with mean, HbA1c, SD, *J*-index, HBGI, ADDR, MAGE, LI, and TAR and negative correlations with MAG and *M* value. OGTT 2-h blood glucose levels at 42 days postpartum showed positive correlations with ADDR, MAGE, LI, and TAR and negative correlations with *M* value. The absolute value change from OGTT results at diagnosis to postpartum OGTT results exhibited correlations with various metrics similar to those seen with postpartum OGTT results alone.

In multivariate logistic regression analysis adjusting for patient age, prepregnancy BMI, and family history of diabetes, LBGI (OR: 1.437; 95% CI: 1.015, 2.035; *p* = 0.041), *M* value (OR: 1.215; 95% CI: 1.030, 1.434; *p* = 0.021), and TBR% (OR: 1.138; 95% CI: 1.020, 1.271; *p* = 0.021) emerged as an independent factor influencing IFG (Table 2). After adjusting for these variables, no GV metrics were identified as independent factors for IGT, although HbA1c% with *p* = 0.053 approached significance.

**Figure 2:**
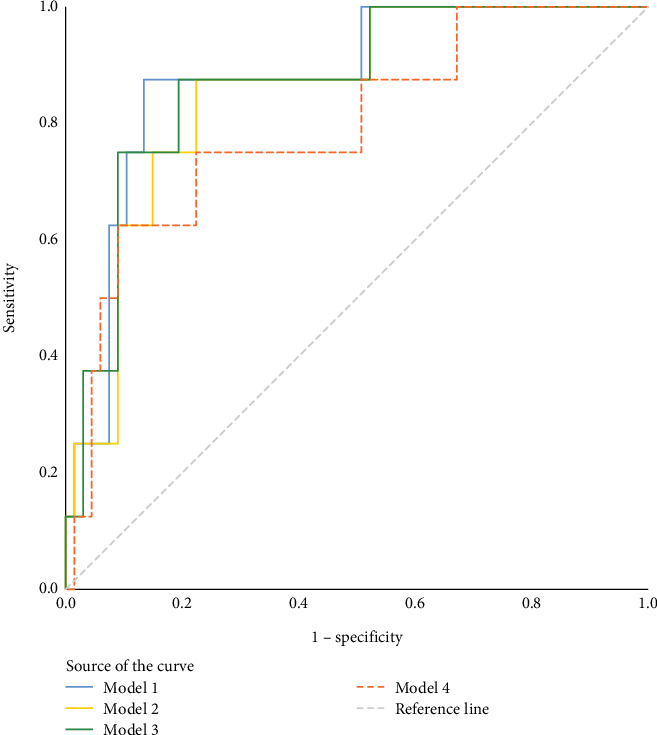
ROC curves of MODEL1, MODEL2, MODEL3, and MODEL4 predicting impaired fasting blood glucose. MODEL1: 0.13∗TBR% + 0.257∗age + (−0.066∗BMI) + (−19.368∗FHD); MODEL2: 0.363∗LBGI + 0.284∗age + (−0.061∗BMI) + (−19.401∗FHD); MODEL3: 0.195∗*M* value + 0.28∗age + (−0.046∗BMI) + (−19.291∗FHD); MODEL4: (−0.122∗HbA1c) + (0.227∗age) + (−0.086∗BMI) + (−19.61∗FHD).

The ROC curve was used to evaluate the prediction model performance of TBR%, LBGI, and *M* value combined with age, BMI, and family history of diabetes, respectively (Figure 2). The area under the curve (AUC) is 0.877 (95% CI: 0.760~0.994), 0.853 (95% CI: 0.730~0.975), 0.869 (95% CI: 0.748~0.991), and 0.793 (95% CI: 0.622~0.963) (Table 3).

## 4. Discussion

Due to effective blood glucose management among our patient population, adverse pregnancy outcomes were infrequent, and we did not observe correlations between various GV indices and adverse pregnancy outcomes or newborn weight. However, prior studies have shown that women with GDM experience greater GV compared to healthy pregnant women, and this variability is associated with an increased risk of adverse pregnancy outcomes [21–23]. High glycemic fluctuations during pregnancy have been identified as the strongest predictor for macrosomia [24], while low blood glucose levels may hinder fetal growth [23]. Besides well-known short-term risks such as macrosomia, preterm birth, preeclampsia, and cesarean delivery [25], women with GDM face an increased long-term risk of developing T2DM. Approximately 60% of women with a history of GDM develop T2DM later in life, with each subsequent pregnancy increasing the risk threefold [26].

Our study indicates that higher maternal GV during pregnancy independently influences the occurrence of IFG at 42 days postpartum, even among those with good glycemic control. Interestingly, different GV metrics showed varying associations with IFG at 42 days postpartum. Metrics related to low blood glucose events such as TBR and LBGI appeared more significantly associated, whereas metrics related to high blood glucose fluctuations such as TAR, HBGI, and other indices like LI and MAGEs correlated more with OGTT 1-h and 2-h glucose levels. Contrary to our initial hypothesis, these GV indices exhibited broader associations with OGTT results at 4–5 months postdiagnosis compared to during CGM monitoring 1 week prior. Considering the temporal sequence, higher GV seems to be the reason of abnormal postpartum OGTT results rather than being a consequence.

A notable finding was that among all GV indicators, only *M* value, TBR%, and LBGI were independent factors of IFG at 42 days postpartum. *M* value represents the average of logarithmic transformations of ratios between observed and ideal reference glucose values (defaulted to 120 mg/dL in EasyGV 9.0), emphasizing the impact of ratios less than 1, particularly focusing on hypoglycemia rather than hyperglycemia [27, 28]. LBGI means frequency and severity of hypoglycemic episodes, and TBR means percentage of time spent below the target glucose range. This may help explain the link between the three. Together, these findings underscore the importance of low blood glucose events in contributing to elevated fasting glucose levels postpartum. Although it is known that hypoglycemic events can elevate blood glucose through pathways such as counterregulatory hormone secretion and inhibition of insulin synthesis and secretion [29], our study shows that even months later, the occurrence of hypoglycemia remains correlated with higher fasting glucose levels. To our knowledge, no other studies have determined the significant impact of hypoglycemic events on long-term fasting glucose levels in pregnant women, and thus, it remains unclear if this conclusion applies uniquely to this population.

Using TIR and TBR% as standards for blood glucose management remains controversial in pregnant women with T2DM, especially those with well-controlled blood glucose [30–32]. Our findings further support the use of CGM and strict limits on TBR in pregnant women with GDM. Larger prospective cohort studies with longer follow-up times are needed to further confirm TBR management thresholds to reduce postpartum diabetes risk.

Compared to women with normal glucose tolerance during pregnancy, women with GDM have a sevenfold increased risk of developing Type 2 diabetes later in life [33]. Due to low attendance at postpartum screening, these patients often do not receive effective treatment in a timely manner [34]. In our study, for example, less than 30% of patients participated in postpartum OGTT screening when contacted by phone and WECHAT, which highlights the importance of risk prediction in advance. Several models have been proposed to predict high-risk populations, with family history of diabetes, prepregnancy BMI, and age considered to be the most commonly used primary predictors [35]. We propose GV as a new predictor, which increases the predictive efficiency of the original model.

Our current study has limitations. Firstly, our study cohort was relatively small, and we did not collect sufficient adverse pregnancy outcome events to establish relevant thresholds for improving pregnancy outcomes. Additionally, given the low number of cases with IFG, caution is warranted when interpreting models assessing the relationship between maternal GV and hypertension during pregnancy. Secondly, our multivariate analysis did not include certain covariates such as maternal lipids and dietary status, crucial indicators of metabolic health.

## Figures and Tables

**Table 1:** Baseline information.

**Indicators**	**Value**
*Population characteristics*	
Age	31.91 ± 3.40
BMI	21.83 ± 2.59
Residence (urban)	85.33% (64)
Occupation	Government-owned enterprises: 42.67% (32) Self-employed individuals: 26.67% (20) Housewives: 9.33% (7) Other: 21.33% (16)
Education	Primary school and below: 0% (0) Secondary vocational school and high school: 4.00% (3) College or associate degree: 65.33% (49) Postgraduate or above: 21.33% (16) Other: 9.33% (7)
Assisted reproduction	8% (6)
*Pregnancy outcomes*	
Mode of delivery	37.33% (28)
Premature birth	0% (0)
Macrosomia	1.33% (1)
Low birth weight infant	1.33% (1)
Neonatal hypoglycemia	1.33% (1)
Preeclampsia	4.00% (3)
*Results of OGTT at 42 days postpartum*	
IFG	10.67% (8)
IGT	9.33% (7)
Diabetes	0% (0)
*Blood glucose status*	
HbA1c%	5.20 (4.99, 5.40)
Mean	5.32 (5.06, 5.64)
*GV metrics*	
CV	0.19 (0.16, 0.23)
SD	1.10 (0.86, 1.24)
*J*-index	13.37 (11.52, 15.17)
LBGI	2.66 (1.86, 3.80)
HBGI	0.86 (0.55, 1.37)
ADRR	2.76 (1.43, 4.91)
GRADE	0.49 (0.37, 0.77)
MAGE	2.26 ± 0.65
MAG	1.66 (1.45, 1.95)
LI	0.86 (0.65, 1.27)
*M* value	3.48 (2.15, 5.65)
CONGA	4.71 (4.52, 5.10)
TIR%	94.33 (90.18, 97.60)
TAR%	2.5 (0.91, 5.39)
TBR%	0.72 (0.18, 3.46)
MODD	0.87 (0.70, 1.05)

**Table 2:** Results of logistic regression analysis adjusting for age, prepregnancy BMI, and family history of diabetes, showing associations of various GV metrics with IFG and IGT.

	**IFG**		**IGT**	
	**OR (95% CI)**	**p** ** value**	**OR (95% CI)**	**p** ** value**
HbA1c%	0.885 (0.097, 8.098)	0.914	1.372 (0.999, 2.406)	0.053
Mean	0.306 (0.057, 1.648)	0.168	0.792 (0.130, 4.826)	0.801
SD	4.145 (0.611, 28.124)	0.146	0.071 (0.001, 3.431)	0.181
*J*-index	1.038 (0.887, 1.217)	0.640	0.895 (0.654, 1.225)	0.490
LBGI	1.437 (1.015, 2.035)	0.041	0.581 (0.224, 1.508)	0.265
HBGI	1.286 (0.839, 1.971)	0.248	0.628 (0.106, 3.719)	0.609
ADDR	0.875 (0.638, 1.200)	0.407	0.890 (0.605, 1.309)	0.553
GRADE	1.738 (0.529, 5.713)	0.362	0.104 (0.004, 2.606)	0.168
MAGE	0.503 (0.124, 2.035)	0.335	0.528 (0.100, 2.803)	0.453
MAG	3.683 (0.825, 16.438)	0.088	0.065 (0.002, 2.029)	0.120
LI	0.594 (0.111, 3.184)	0.543	0.757 (0.107, 5.373)	0.780
*M* value	1.215 (1.030, 1.434)	0.021	0.741 (0.436, 1.262)	0.270
CONGA	0.920 (0.241, 3.517)	0.903	0.656 (0.090, 4.763)	0.677
TIR%	0.942 (0.868, 1.022)	0.153	1.133 (0.942, 1.364)	0.186
TAR%	0.905 (0.737, 1.111)	0.339	0.936 (0.765, 1.145)	0.520
TBR%	1.138 (1.020, 1.271)	0.021	0.729 (0.407, 1.304)	0.286
MODD	3.402 (0.227, 50.887)	0.375	0.004 (0.000, 2.074)	0.083

Abbreviations: IFG, impaired fasting glucose; IGT, impaired glucose tolerance.

**Table 3:** ROC statistical results for MODEL1, MODEL2, MODEL3, and MODEL4 predicting impaired fasting blood glucose.

**Assessment methods**	**AUC (95% CI)**	**p**	**Sensitivity**	**Specificity**	**Youden's index**	**Cut-off value**
MODEL1	0.877 (0.760~0.994)	0.001	0.875	0.866	0.741	0.140
MODEL2	0.853 (0.730~0.975)	0.001	0.875	0.776	0.651	0.125
MODEL3	0.869 (0.748~0.991)	0.001	0.875	0.806	0.681	0.125
MODEL4	0.793 (0.622~0.963)	0.007	0.625	0.910	0.535	0.212

*Note:* MODEL1: 0.13∗TBR% + 0.257∗age + (−0.066∗BMI) + (−19.368∗FHD); MODEL2: 0.363∗LBGI + 0.284∗age + (−0.061∗BMI) + (−19.401∗FHD); MODEL3: 0.195∗*M* value + 0.28∗age + (−0.046∗BMI) + (−19.291∗FHD); MODEL4: (−0.122∗HbA1c) + (0.227∗age) + (−0.086∗BMI) + (−19.61∗FHD).

Abbreviation: FHD, family history of diabetes.

## Data Availability

Research data are not shared.
